# Essential Oils of New *Lippia alba* Genotypes Analyzed by Flow-Modulated Comprehensive Two-Dimensional Gas Chromatography (GC×GC) and Chemometric Analysis

**DOI:** 10.3390/molecules26082332

**Published:** 2021-04-16

**Authors:** Leila Gimenes, Júlio César R. Lopes Silva, Roselaine Facanali, Leandro Wang Hantao, Walter José Siqueira, Marcia Ortiz Mayo Marques

**Affiliations:** 1 Vegetable Genetic Resources Center, Agronomic Institute, Campinas 13075-630, Brazil; juliocesarls2009@hotmail.com (J.C.R.L.S.); siqueirawj@gmail.com (W.J.S.); 2 School of Agriculture, São Paulo State University (Unesp), Botucatu 18610-034, Brazil; 3 Institute of Chemistry, University of Campinas (Unicamp), Campinas 13083-970, Brazil; roselainefacanali@gmail.com (R.F.); wang@unicamp.br (L.W.H.)

**Keywords:** *Lippia alba*, lemon balm, essential oils, flow-modulated, comprehensive two-dimensional gas chromatography

## Abstract

*Lippia alba* (Mill.) N. E. Br. (Verbenaceae) is an aromatic shrub whose essential oils have stood out as a promising source for application in several industrial fields. In this study, the essential oils chemical characterization of eight new *L. alba* genotypes was performed. The selected materials were collected from the Active Germplasm Bank of the Agronomic Institute and the essential oils were extracted by hydrodistillation. Flow-modulated comprehensive two-dimensional gas chromatography coupled to mass spectrometry (GC×GC-MS) was employed for chemical characterization and evaluation of possible co-eluted compounds. In addition, the chemical analyses were submitted to multivariate statistical analyses. From this investigation, 73 metabolites were identified in the essential oils of the genotypes, from which α-pinene, β-myrcene, 1,8-cineole, linalool, neral, geranial, and caryophyllene oxide were the most abundant compounds among the accessions. This is the first report disclosing α-pinene in higher amounts in *L. alba* (19.69%). In addition, sabinene, *trans*-verbenol, myrtenol, (*E*)-caryophyllene, α-guaiene, germacrene D, and α-bulnesene were also found in relevant quantities in some of the genotypes, and myrtenal and myrtenol could be well separated through the second dimension. Such results contributed to the understanding of the chemical composition of those new genotypes, being important to drive a future industrial applicability and studies in genetic breeding.

## 1. Introduction

*Lippia alba* (Mill) N. E. Brown (Verbenaceae) is a vigorous and rustic shrub originated from South and Central Americas, occurring with wide distribution in Brazil. The species, which is popularly known as lemon balm, is largely used in folk medicine, characterized as being one of the most important medicinal plants used in Brazil [1,2]. The species is consumed fresh, being prepared in the form of teas, sweets, extracts, syrups, and tinctures. Tea preparations of its leaves, for example, are popularly employed in the treatment of gastrointestinal, diarrhea and dysentery disorders and as having tranquilizing, sedative, and analgesic actions [1,3]. Moreover, as an aromatic plant, its essential oils (EOs) have also been used in preparations of cosmetics, perfumes, and hygiene products, already available to consumers [4].

*L. alba* is a plant that presents great agronomic potential, with rapid and aggressive development and easy cultivation [2]. All the features previously mentioned have stood out the species as a promising plant for an economic exploration that might be applied as a natural source with different purposes and in varied fields, such as pharmaceutical, cosmetic, perfumery and agricultural industries [5]. However, as higher is the demand of compounds extracted from natural resources, the higher is the over-exploitation of native species that is also threatened by habitat transformation, pollution, and climate changes, endangering the extinction of the same. This is the case of some species of *Lippia* genus from Minas Gerais and Goias states (Cadeia do Espinhaço region) in Brazil, that are at risk of extinction due the vulnerability of that region [6]. Hence, to reach the sustainable cultivation of species with economic potential, genetic resources maintained in germplasm banks have been established as a fundamental role for biodiversity conservation and to provide genetic diversity [7,8,9]. In addition, studies of genetic breeding programs are important for the standardization of the chemical phenotype and can be used to select the well adapted and potential genotypes of the existing germplasm, improving quality and productivity as well as developing varieties with enhanced value to the market [1,9,10]. These researches become a powerful complement to fulfill the worldwide demand for quality products besides being a strategic tool for the development of new raw materials for industrial use (pharmaceutical, perfumery, and beverages) [9,10].

Previous studies of *L. alba* reported the evaluation of genetic parameters such as performance for environment stability, adaptability, and Genotype × Environment interactions. These studies resulted in a bank of aromas and fragrances with over 100 new combinations of plants belonging to the Agronomic Institute (IAC) in Campinas, Brazil [5,11], affording relevant information that subsequently led to the genetic breeding program from IAC to perform biparental crosses of the most promisor cultivars, aiming to obtain plants with different genetic variability. Hence, crossings between the genotypes linalool, myrcene/camphor, limonene/camphor, and citral resulted in new hybrids with different combinations of major constituents [11,12]. As far as we know, it was the first genetic breeding of *L. alba* in Brazil and worldwide. However, none of these materials were evaluated regarding their chemical composition so far.

In addition, although the species has been extensively a target of scientific studies regarding its chemical composition and evaluation of its biological profiles [13], a deeper investigation of its constituents and analysis of possible co-eluted compounds (quite common observed in natural matrices) is still lacking in studies. A previous study with the plant by Multidimensional Gas Chromatography (MDGC) showed the evaluation of the enantiomeric ratios of α-pinene, sabinene, limonene, and linalool. However, it was described the investigation of only one genotype and the employment of a heart-cut system of the enantiomeric fractions instead of the full sample analysis [14].

Hence, comprehensive analyses of the full sample are essential to reach the requirements of quality assurance, safety, and efficacy of a product, once it makes possible to discriminate beneficial compounds, as well as monitoring important parameters as adulterations and variability prior to the development for a subsequent commercialization of any natural formulations [15]. Moreover, a comprehensive identification of the substances in the sample can also contribute to the understanding of synergistic or antagonistic effects that might mediate some relevant biological activity [16].

Analytical tools that provide high resolution and sensitivity for untargeted analysis of the plant metabolome are desperately needed. Among these tools, comprehensive two-dimensional gas chromatography coupled to mass spectrometry (GC×GC-MS) is characterized for the increased peak capacity [17,18,19], increasing the identification power of plants volatiles and semi-volatiles composition [20,21].

Additionally, chemometrics is a versatile and useful tool to explore the diversity of metabolic profile in a dataset, being helpful to retrieve more valuable chemical information from natural matrices due the combination of mathematics, statistics, and computer science [22,23]. Hence, the combination of the GC×GC-MS with multivariate analysis provides a better information on the similarity and relationship among the EOs chemical composition, enabling the visualization of the clustering patterns within the samples [24,25].

Thus, the objective of this work was to perform the chemical characterization of new genotypes combinations obtained from biparental crosses of *L. alba* by flow-modulated GC×GC-MS (FM-GC×GC-MS) combined to multivariate data analyses using principal component analysis (PCA) and hierarchical cluster analysis (HCA). This is the first investigation of *Lippia* species employing this modern technique. This study aims to contribute for a well understanding of these new plant crossings that might represent materials with highlighted agronomic value, as well as driving future genetic breeding studies and consequently obtention of different combinations of chemical phenotype for a future industrial applicability.

## 2. Results and Discussion

### Extraction Yield and Chemical Composition of the Essential Oils

The hydrodistillation of the leaves of the eight studied genotypes yielded in yellowish oils with characteristics of citrus odor. The yields of volatiles oils from the leaves of each genotype ranged from 0.29 to 1.03%, as described in Table 1. The highest yield was found in the X6MIA genotype (1.03%), whereas X6M6 genotype disclosed the lowest yield (0.29%).

**Figure 1:**
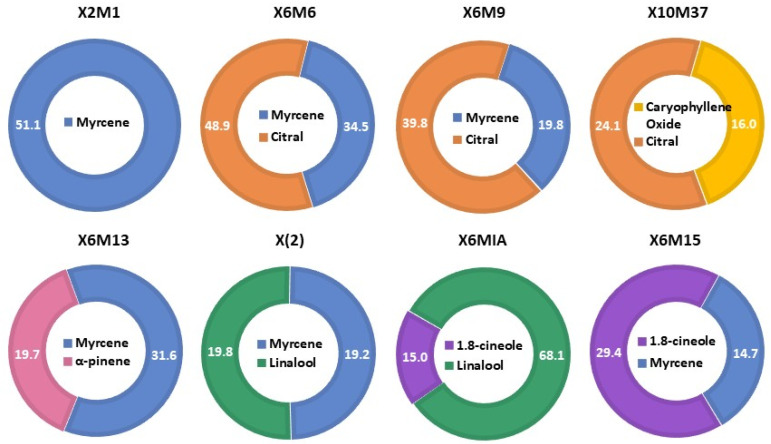
Major compounds and its relative percentage (%) in the genotypes of *L. alba*.

**Figure 2:**
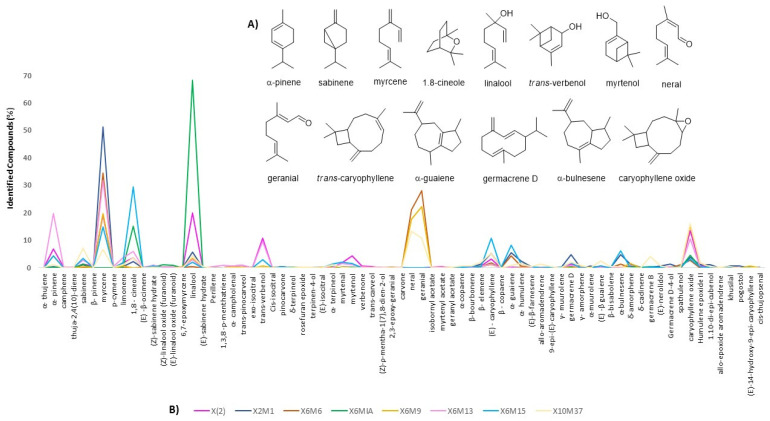
(**A**) Structure of the main compounds found in the new genotypes of *L. alba*. (**B**) Identified compounds and comparison of their distribution (relative abundance) among all genotypes.

The analysis of the essential oil chemical composition from eight genotypes of *L. alba* allowed the identification of 73 secondary metabolites, as disclosed in Table 2. In general, the constituents identified in oils belong to the terpenoids class, with the following predominance: monoterpene hydrocarbons (1.53–59.79%) and oxygenated monoterpenes (9.99–86.44%), sesquiterpene hydrocarbons (3.07–30.49%), and oxygenated sesquiterpenes (3.96–19.49%), from which α-pinene, β-myrcene, 1,8-cineole, linalool, citral (neral + geranial), and caryophyllene oxide were the most abundant compounds among the accessions, enabling the separation of the genotypes into different chemotypes of *L. alba* Figure 1. In addition, an overlap of the chemical composition of all genotypes displayed the compounds sabinene, *trans*-verbenol, myrtenol, (*E*)-caryophyllene, α-guaiene, germacrene D, and α-bulnesene in relevant quantities in some of the genotypes, as disclosed in Figure 2A (chemical structures) and Figure 2B (identified compounds in the essential oils). The relative percentage of all identified compounds is shown in Table 2.

The essential oil of the X2M1 genotype showed the highest abundance of β-myrcene (51.11%), also observed to the genotypes X6M6 (34.50%) and X6M13 (31.59%, Figure 1), and the values described in this work are higher than those that are commonly reported in literature to this species, ranging mainly from 0.30 to 25.81% among the distinct chemotypes analyzed from different geographical origin [28,29,30,31]. Marques, (2018), e.g., observed an increase of 1.30% of the production of β-myrcene after cultivation of the plant in green manures in succession [32]. However, the highest abundance of this compound reached only 8.04%. A second work disclosed 15.00% of β-myrcene of a myrcene-citral chemotype of *L. alba* [33]. Our results, however, were similar to that reported by Jannuzzi, (2010) [34], which one among the sixteen analyzed chemotypes revealed the abundance of 47.60% of β-myrcene. Additionally, our findings are also comparable to those matrices already known of having higher quantities of this compound, as, e.g., in hops essential oils. Studies of the essential oils of hop Polish cultivars afforded from 29.90 to 67.00% of this monoterpene [35].

In a general way, β-myrcene was present in all analyzed accessions of *L. alba*, except in X6MIA. β-myrcene is a colorless or light-yellow oily monoterpene with an important industrial value. Due to its pleasant smell with a woody, spicy, peach, sweet, vanilla, and wine-like odor description, this compound is a value intermediate for the preparation of flavor and fragrance chemicals. Furthermore, myrcene is also described as a versatile starting material for vitamins and pharmaceuticals due to its reactive diene structure [36,37,38,39], and as having significant pharmacological properties, such as anti-inflammatory and anti-catabolic effects for deceleration of osteoarthritis progression [40], and as an activator for TRPV1 as target for treating pain [41].

The essential oil of the X6M13 genotype in addition to β-myrcene also showed a high abundance of α-pinene (31.59% and 19.69%, respectively, Figure 1). Usually, the abundance of α-pinene in *L. alba* essential oils, when reported, is extremely low, reaching less than 1.0% [1,33,42]. Our results pinpoint to a new myrcene-α-pinene chemotype. However, further seasonality investigations are still needed to confirm this chemotype. α-pinene is a colorless and water-insoluble volatile plant metabolite, found in many essential oils and being the major monoterpene of pine trees. As a safe food additive approved by the U.S. Food and Drug Administration, this compound has been widely used as a food-flavoring ingredient [43,44]. In addition, several biological activities have been attributed to α-pinene, including antibacterial and antifungal [45], apoptotic and antimetastatic effect [46], anti-inflammatory and chondroprotective [47], and gastroprotective [48] effects.

The essential oil of the X6M6 and X6M9 genotypes in addition to β-myrcene, also showed a high abundance of citral (neral + geranial) as their major composition. The X10M37 genotype, however, disclosed citral and caryophyllene oxide as its major components Figure 1. The chemotype citral in *L. alba* is very well established, and in general, its abundance is reported above 65.00% [1,49]. Studies regarding the chemical composition of myrcene-citral chemotype, on the other hand, have shown an abundance of these compounds similar to our results, ranging 13.00–15.00% of myrcene and 37–53% of citral [33,34]. Citral is an open chain monoterpenoid formed by the neral and geranial stereoisomers found in several medicinal plants and widely used as additives in foods, beverages, and cosmetics, due its intense lemon aroma and flavor [50]. A plethora of pharmacological properties have already been reported to citral, such as antibacterial [51], antifungal [52], insecticide [53], antioxidant [16], anticancer [54], anti-inflammatory [55], and anti-nociceptive [56] activities.

The essential oil of the X6MIA genotype disclosed linalool and 1,8-cineole as its major constituents (68.10% and 15.0%, respectively, Figure 1). Similar results of these compounds were also described by Barros, 2009 (63.70% of linalool and 10.40% of 1,8-cineole) [57]. The genotype X(2), however, disclosed linalool and myrcene (19.80% and 19.20%, respectively) as its major compounds Figure 1. Linalool is an acyclic monoterpene alcohol widely used in cosmetics and flavoring ingredients [58]. Moreover, previous works have demonstrated linalool to have a comprehensive range of biological properties, such as potent antibacterial [59], anti-inflammatory [60,61], antioxidant, anticancer [62], antinociceptive [58], anxiolytic, and neuroprotective [63] agent.

The essential oil of the X6M15 genotype disclosed 1,8-cineole and myrcene as its major constituents (29.40 and 14.70%, respectively, Figure 1). The existence of a 1,8-cineole:myrcene chemotype was reported by Ricciardi, 2009 [31], in which the amounts of these compounds reached only 14.70% and 10.40%, respectively.1,8-cineole is a saturated monoterpene with pleasant aroma and taste, widely used in food, fragrances, and cosmetics [64]. Plenty studies have reported the substances as having benefits for respiratory tract infection, such as bronchitis, sinusitis as well as exhibiting secretolytic and bronchospasmolytic properties, and anti-inflammatory, antimicrobial, and antiseptic efficacy [64].

**Figure 3:**
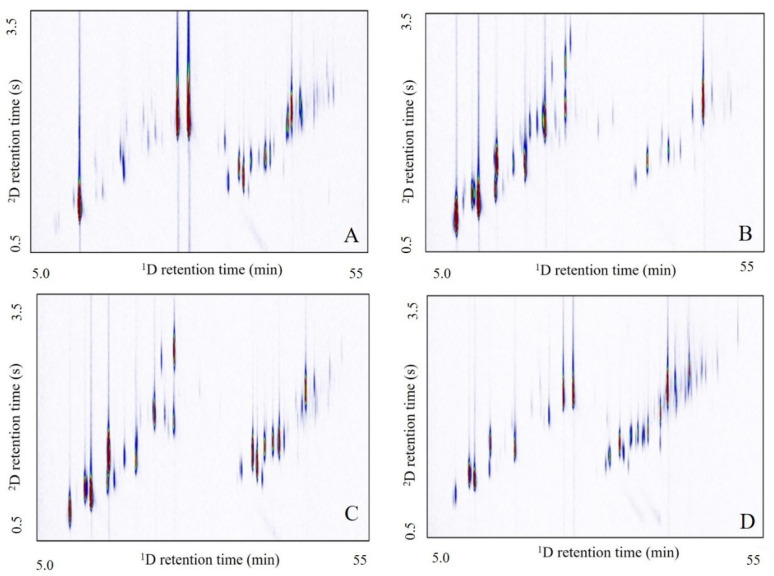
GC×GC-MS total ion chromatograms (TIC) of essential oils of new *L. alba* genotypes: (**A**) X6M6, (**B**) X6M13, (**C**) X6M15, and (**D**) X10M37.

**Figure 4:**
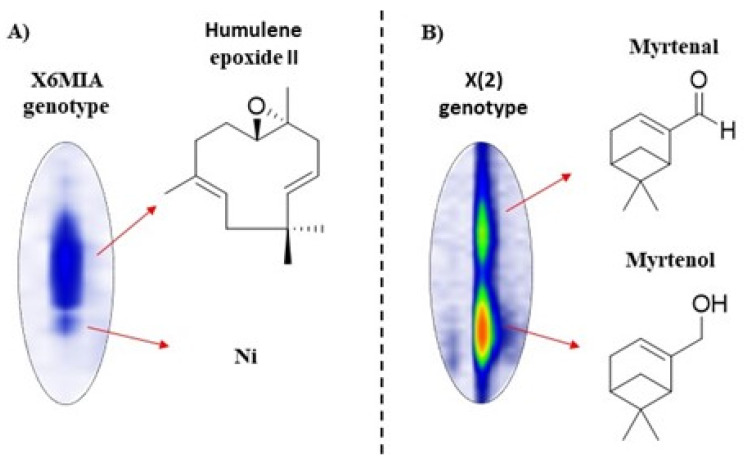
GC×GC-MS contour plots showing examples of improved peak resolution of two overlapping clusters. (**A**) Coelution between humulene epoxide II (RT1D = 42.33; RT2D = 2.63 s) and an unknown compound (Ni = not identified compound; RT1D = 42.33; RT2D = 2.44 s), (**B**) Coelution between myrtenal (RT1D = 23.67; RT2D = 2.99 s) and myrtenol (RT1D = 23.67; RT2D = 2.45 s).

Although major constituents present in the genotypes might be effective to drive a future biological application of the samples, it should be noted that different ratios of these components can affect the final behavior of the same. In addition, a deeper investigation and knowledge of other minor constituents are pivotal since synergistic or antagonistic effects might influence the overall performance [16,65]. Into this perspective, the analyses of the *L. alba* essential oils allowed the identification of 26 compounds in low concentrations (up to 0.1%) at least in one of the eight analyzed genotypes, considered as trace. In addition, 66 compounds were identified in concentrations up to 0.5% in at least one of the eight genotypes Table 2. The typical GC×CG-MS total ion chromatograms for the most representative essential oils are disclosed in Figure 3. In addition, it is also relevant the evaluation of possible co-eluted components in the samples for their chemical composition discrimination. Hence, the analyses of the GC×GC-MS allowed to verify the presence of co-eluted compounds in five genotypes. Whereas the coelution of humulene epoxide II with a second unknown compound Figure 4A was noticed to the X6MIA genotype, myrtenal and myrtenol were well separated and identified in the X(2), X2M1, X6M13, and X6M15 genotypes Figure 4B.

In order to investigate the similarity and relationship among the EOs chemical composition of *L. alba* genotypes, hierarchical cluster analysis (HCA) and principal component analysis (PCA) were constructed to the oil components. From the HCA analysis, it was observed a separation among the samples, which the X(2) and X6M13 genotypes (group I) were the most dissimilar, followed by the X10M37 genotype (group II). The remaining genotypes (X6M6, X6M9, X6M15, and X6MIA, group III) showed a greater chemical similarity to each other.

For PCA analysis, a three-component PCA model expressed 63.67% of the total variance, with the first principal component (PC1) being responsible for 27.62%, the second principal component (PC2) for 19.46% and the third principal component (PC3) for 16.59% of the total variance, making it possible to determine the most significant substances in the data set.

Thus, PC1 positively correlated to α-pinene (**2**), found in a higher relative proportion in the X6M13 genotype, camphene (**3**), β-pinene (**6**), *trans*-pinocarveol (**21**), *trans*-verbenol (**23**) and isobornyl acetate (**40**), and negatively correlated to (*E*)-β-farnesene (**50**), *allo*-aromadendrene (**51**), 9-*epi*-(*E*)-caryophyllene (**52**), β-bisabolene (**58**), γ-amorphene (**55**), (*E*)-β-guaiene (**57**), germacrene B (**62**), and *allo*-epoxide aromadendrene (**69**). PC2, in addition, positively correlated sabinene (**5**), *p*-cymene (**8**) and β-bourbonene (**44**) and negatively correlated to γ-muurolene (**53**) and δ-cadinene (**61**). PC3, on the other hand, showed positive correlations to germacrene D (**54**), *cis*-thujopsenal (**73**), pogostol (**71**), germacrene D-4-ol (**64**), α-bulnesene (**59**), α-humulene (**49**), and pinocarvone (**25**), and a negative correlation to (*E*)-14-hydroxy-9-*epi*-caryophyllene (**72**), neral (**37**), geranial (**39**), and 6,7-epoxymyrcene (**15**).

**Figure 5:**
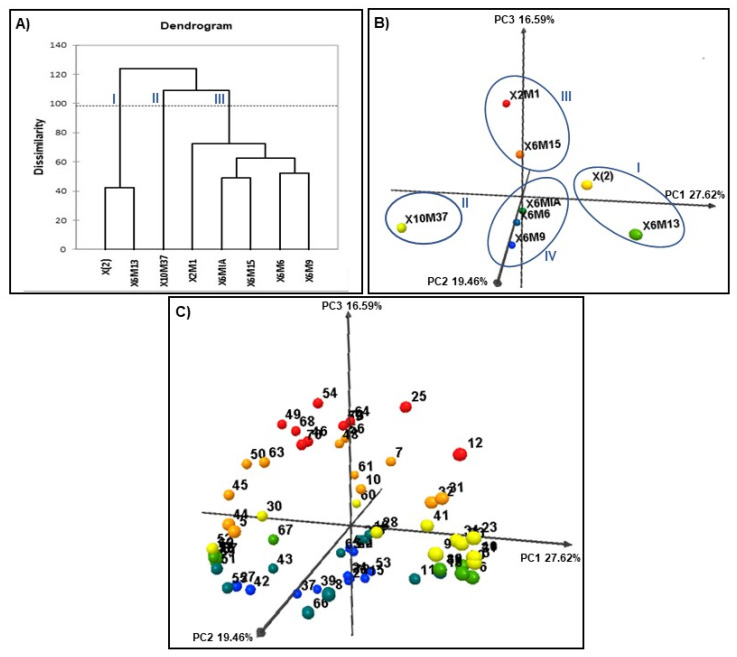
Dendrogram (**A**) obtained from HCA, scores plot (**B**) and loadings plot (**C**) obtained from PCA of the chemical composition data of the *L. alba* essential oils. Numbers in loadings plot (**C**) represent the identified compounds as described in Table 2.

The dendrogram from the HCA analysis is illustrated in Figure 5A, whereas the scores graph from the PCA analysis is illustrated in Figure 5B. The compounds responsible for the observed clustering among the samples is disclosed in Figure 5C.

These analyses allowed to distinguish the samples in four main groups: (X(2) and X6M13, group I), (X10M37, group II), (X6M15 and X2M1, group III), and (X6M6, X6M9, and X6MIA, group IV). This classification had been previously supported by the dendrogram from the HCA analysis, mainly allowing the distinction of the groups I and II.

However, a greater information could be observed from the PCA analysis, which resulted in an efficient subdivision of the group III (previously obtained from HCA) in two groups (III and IV, Figure 5B). A higher similarity between the genotype X2M1 and X6M15 was observed Figure 5B and the compounds α-guaiene (**48**), α-bulnesene (**59**), α-humulene (**49**), germacrene D (**54**), and (*E*)-caryophyllene (**46**) could be pinpointed from the PCA biplot graph as the responsible for such similarities Figure 5C. Indeed, those genotypes disclosed the highest percentage of sesquiterpenes hydrocarbon among the samples Table 2.

## 3. Materials and Methods

### 3.1. Plant Material Collection

Leaves of eight *L. alba* genotypes coded as X(2), X2M1, X6M6, X6MIA, X6M9, X6M13, X6M15, and X10M37 were collected from the germplasm bank located at Agronomic Institute (IAC), Campinas, Brazil (22°54′ latitude S and 47°05′ longitude W). The branches were collected in January 2018 and the leaves were manually separated from the branches and subsequently dried in an oven with air circulation at 40 °C for 48 h up to constant weight.

### 3.2. Essential Oils Extraction

About 80 g of the dried leaves from each *L. alba* genotype were submitted to a hydrodistillation using Clevenger apparatus for two hours. Obtained essential oils were then separated from aqueous phase and kept in sealed vials at −20 °C in the dark for further analysis. The yield (%) of EOs was calculated based on plant material dry in grams.

### 3.3. Essential Oil Chemical Characterization

Samples were diluted in ethyl acetate (Tedia, chromatographic grade, Fairfield, OH, USA) at the concentration of 1 mg/mL and 1 µL of each solution was injected.

The GC×GC-QMS experiments were conducted on a TRACE 1310 gas chromatograph coupled to a fast-scanning ISQ single transmission quadrupole mass spectrometer (MS) (Thermo Scientific—Waltham, MA, USA). The GC was equipped with a split/splitless injector (SSL) (ThermoFisher Scientific—Austin, TX, USA). A Topaz 4.0 mm-id split precision inlet liner (Restek Corporation—Bellefonte, PA, USA) was used for sample vaporization. A TriPlus RSH autosampler (ThermoFisher Scientific) fitted with a 10 µL syringe (Trajan Scientific—San Diego, CA, USA) was used to inject the liquid sample. The rinsing solvents were isopropanol and methylene chloride.

Differential flow modulation was performed in reverse fill/flush (RFF) configuration using the INSIGHT flow modulator (SepSolve Analytical—Waterloo, ON, Canada). ChromSpace software (1.9 version, SepSolve Analytical) was used to control the INSIGHT modulator. Instrument control and data acquisition were performed using Xcalibur software (ThermoFisher Scientific). The column arrangement consisted of two wall-coated open tubular (WCOT) capillary columns. The primary column was a 30 m × 0.25 mm-id × 0.25 μm (β = 250) HP-5MS (Agilent Technologies—Santa Clara, CA, USA). Secondary column was a 5.0 m × 0.25 mm-id × 0.25 μm (β = 250) HP-50+ (Agilent Technologies). A 23 cm × 0.53 mm-id sampling loop of 50 µL (part N. 70058) (Restek Corporation) and a 3.0 m × 0.10 mm-id deactivated fused silica restrictor were used for RFF. An unpurged SilFlow GC 3-port splitter (part N. 123725) (Trajan Scientific) was used for passive division of the secondary column effluent for parallel detection (MS/FID). Two 5.0 m × 0.18 mm-id and 5.0 m × 0.32 mm-id deactivated capillaries (Restek Corporation) were used to connect the three-way splitter to the MS and FID, respectively. A reproducible division of approximately 1:6 was achieved throughout the experiments. The interested reader is directed elsewhere for more details on the instrument setup [17,18,19].

A modulation period of 5 s with a re-injection (flush) pulse of 200 ms was used in all FM-GC×GC analyses. Ultrapure Helium was used as carries gas and auxiliary gas at constant flow rates of 1 mL/min and 20.0 mL/min, respectively. The GC inlet was kept at 250 °C and operated with a split ratio of 1:20. The oven temperature was programed from 60 to 240 °C at 3 °C min^−1^. The ion source and MS transfer line were kept at 220 °C and 250 °C, respectively. Electron ionization was performed at 70 eV and 150 µA emission current. The mass range was set from 45 to 400 Da at 42 scans s^−1^.

Data processing was performed using GC Image software (2020r1.2 version, Zoex—Houston, TX, USA). Compounds were tentatively identified comparing the substance mass spectra with NIST 14 database (National Institute of Standards—Gaithersburg, MD, USA) and filtered by the retention index (LTPRI), adopting minimum similarity match of 80% from NIST and ± 25 LTPRI deviation as those reported by Adams (2017) [26]. GC retention index of each compound was calculated based on injection of a homologous series of C_8_–C_20_ *n*-alkanes (Merck-St. Louis, MO, USA) using the Van den Dool and Kratz equation [27], and the concentration of the metabolites were obtained by area normalization.

### 3.4. Statistical Analyses

The results of the chemical analyses were submitted to multivariate statistical analyses, such as principal components analysis (PCA) and hierarchical clustering analysis (HCA). The models were built using the software XLSTAT-2020 version (Addinsoft—Bordeaux, France).

## 4. Conclusions

In this work, the chemical characterization of eight essential oils from new *L. alba* genotypes was performed by GC×GC-MS combined to multivariate data analyses. From this investigation, the new genotypes could be distinguished in four main groups according to the variance observed through the PCA analysis, indicating a variation in their chemical composition. Higher amount of β-myrcene was observed to the X2M1 genotype, indicating this accession as a natural source to obtain this compound. In addition, higher amounts of sesquiterpenes were observed in the genotypes X2M1 and X6M15. This is the first report disclosing α-pinene in higher amounts in *L. alba* (19.69%, X6M13 genotype), suggesting the existence of a new α-pinene/myrcene chemotype. The use of GC×GC-MS for *L. alba* essentials oils was reported in this work by the first time, allowing the identification of 73 metabolites in the accessions, and the technique was also efficient to identify compounds in low abundance, which 26 compounds were identified in concentrations up to 0.1% in at least one of the eight analyzed genotypes. Furthermore, the compounds myrtenal and myrtenol could be well separated through the second dimension, whereas humulene epoxide II could be identified and well separated from a second unknown compound. The results found in this work contributed to the understanding of the chemical composition of new *L. alba* genotypes and highlight the relevance of these materials as natural sources for a future flavor, fragrance, and pharmaceutical applicability. In addition, this work also emphasizes the relevance of genetic breeding studies to potentially improve the essential oils quality and obtention of different combinations of chemical phenotype.

## Figures and Tables

**Table 1:** Yield of EOs of the eight studied *L. alba* genotypes.

	X(2)	X2M1	X6M6	X6MIA	X6M9	X6M13	X6M15	X10M37
Extraction Yield (%)	0.42	0.38	0.29	1.03	0.30	0.54	0.47	0.57

**Table 2:** Chemical composition of essential oils from eight new *L. alba* genotypes analyzed by GC×GC-MS.

Compound ^a^		LTPRI ^b^			Relative Content (%) ^f^							
		Lit.^c^	Exp.^d^	Similarity (%) ^e^	X(2)	X2M1	X6M6	X6MIA	X6M9	X6M13	X6M15	X10M37
**1**	α-thujene	924	920	92	-	-	-	-	-	-	-	0.24
**2**	α-pinene	932	926	97	6.91	0.27	-	0.16	4.32	19.69	4.36	1.02
**3**	camphene	946	941	90	0.16	-	-	-	-	0.24	-	-
**4**	thuja-2,4(10)-diene	953	947	87	-	-	-	-	-	0.12	-	-
**5**	sabinene	969	965	94	1.35	1.17	0.11	1.37	0.33	2.62	3.43	7.10
**6**	β-pinene	974	968	94	0.60	-	-	-	0.24	0.75	-	-
**7**	myrcene	988	981	95	19.17	51.11	34.5	-	19.79	31.59	14.87	6.57
**8**	*p*-cymene	1020	1015	92	-	-	0.05	-	-	0.27	-	0.21
**9**	limonene	1024	1018	95	1.79	0.31	-	-	0.71	3.55	1.71	1.07
**10**	1,8-cineole	1026	1021	95	3.60	2.15	-	15.05	-	5.95	29.36	3.58
**11**	(*E*)-β-ocimene	1044	1040	92	-	-	0.10	-	-	0.11	-	-
**12**	(*Z*)-sabinene hydrate	1065	1055	86	0.87	0.57	-	0.17	0.12	0.81	0.53	0.10
**13**	(*Z*)-linalool oxide (furanoid)	1067	1061	92	0.15	-	-	1.13	-	-	-	-
**14**	(*E*)-linalool oxide (furanoid)	1084	1077	87	0.10	-	-	0.79	-	-	-	-
**15**	6,7-epoxymyrcene	1090	1080	84	-	0.10	0.21		0.42	0.14	-	-
**16**	linalool	1095	1086	93	19.83	5.59	0.28	68.15	3.39	2.83	2.02	4.28
**17**	*(E)*-sabinene hydrate	1098	1092	80	-	-	-	-	-	-	-	0.37
**18**	perillene	1102	1095	*	-	-	0.06	-	-	0.40	-	-
**19**	1,3,8-*p*-menthatriene	1108	1100	80	-	-	-	-	-	0.85	-	-
**20**	α-campholenal	1122	1114	88	0.50	0.11	-	-	0.30	0.50	-	-
**21**	*trans*-pinocarveol	1135	1129	89	0.77	0.09	-	-	0.18	0.83	-	-
**22**	*exo*-isocitral	1140	1132	80	-	-	0.11	-	-	-	-	-
**23**	*trans*-verbenol	1140	1134	93	10.71	0.42	-	0.20	0.68	9.55	2.88	0.17
**24**	(*Z*)-isocitral	1160	1150	83	-	-	-	-	0.13	-	-	-
**25**	pinocarvone	1160	1152	87	0.40	0.48	-	-		0.27	0.48	-
**26**	δ-terpineol	1162	1155	81	-	-	-	0.18	-	-	-	-
**27**	rosefuran epoxide	1173	1161	80	-	-	-	-	0.10	-	-	0.14
**28**	terpinen-4-ol	1174	1166	85	0.20	-	-	0.17		0.09	0.12	0.11
**29**	(*E*)-isocitral	1177	1168	85	-	-	0.08	-	0.25	-	-	-
**30**	α-terpineol	1188	1179	91	-	-	-	0.44	0.13	-	1.20	1.06
**31**	myrtenal	1194	1184	94	1.84	0.28	-	-	0.46	1.47	1.91	0.10
**32**	myrtenol	1195	1184	94	4.23	0.20	-	0.16	-	1.15	1.52	-
**33**	verbenone	1204	1197	89	0.64	-	-	-	-	0.44	-	-
**34**	*trans*-carveol	1215	1205	82	0.32	-	-	-	-	0.20	-	-
**35**	(*Z*)-*p*-mentha-1(7),8-dien-2-ol	1227	1220	80	-	-	-	-	-	0.10	-	-
**36**	2,3-epoxy-geranial	1234	1224	80	-	-	0.06	-	-	-	-	-
**37**	neral	1235	1225	93	-	-	21.04	-	17.53	-	-	13.34
**38**	carvone	1239	1232	80	-	-	-	-	-	0.10	-	-
**39**	geranial	1264	1254	93	-	-	27.89	-	22.24	-	-	10.81
**40**	isobornyl acetate	1283	1271	80	0.13	-	-	-	-	0.15	-	-
**41**	myrtenyl acetate	1324	1309	86	0.34	-	-	-	-	0.10	-	-
**42**	α-copaene	1374	1367	91	0.08	0.05	-	0.28	0.46	0.04	0.05	0.61
**43**	geranyl acetate	1379	1370	80	-	-	0.21	-	-	-	-	0.18
**44**	β-bourbonene	1387	1376	87	0.13	0.09	0.05	0.04	0.05	0.14	0.32	0.56
**45**	β-elemene	1389	1380	92	0.59	1.28	0.39	0.38	0.56	0.28	0.33	1.99
**46**	(*E*)-caryophyllene	1417	1411	94	3.20	5.12	1.67	1.22	1.03	1.29	10.8	5.11
**47**	β-copaene	1430	1421	83	-	0.08	-	0.09	0.19	-	-	-
**48**	α-guaiene	1437	1429	93	0.14	5.42	4.22	-	-	-	8.26	0.80
**49**	α-humulene	1452	1446	92	0.18	2.33	0.53	0.18	0.21	-	1.51	1.45
**50**	(*E*)-β-farnesene	1454	1438	87	-	0.38	0.11	-	0.18	-	0.37	0.46
**51**	*allo*-aromadendrene	1458	1454	88	0.08	-	-	0.11	0.16	0.10	-	1.27
**52**	9-*epi*-(*E*)-caryophyllene	1464	1460	90	-	-	-	-	-	-	0.09	0.40
**53**	γ-muurolene	1478	1465	88		-		0.10	0.09	0.05	0.05	-
**54**	germacrene D	1480	1473	93	1.19	4.70	0.12	0.48	-	0.83	1.84	1.89
**55**	γ-amorphene	1495	1494	86	0.07	-	-	0.10	0.37	0.10	-	0.83
**56**	α-muurolene	1500	1495	85	-	0.49	-	0.11	0.11	-	-	-
**57**	(*E*)-β-guaiene	1502	1497	80	-	-	0.57	-	-	0.24	0.54	2.46
**58**	β-bisabolene	1505	1500	80	-	-	-	-	-	-	-	0.47
**59**	α-bulnesene	1509	1502	91	0.12	4.80	1.23	-	-	-	6.22	0.28
**60**	δ-amorphene	1511	1504	83	0.53	1.26	-	0.36	1.61	-	-	-
**61**	δ-cadinene	1522	1510	84	-	0.10	-	0.12	-	-	-	-
**62**	germacrene B	1559	1549	90	-	0.19	-	0.32	0.08	-	0.11	3.94
**63**	(*E*)-nerolidol	1561	1551	88	0.32	0.50	0.05	0.14	0.15	0.06	0.07	0.52
**64**	germacrene D-4-ol	1574	1563	80	0.23	1.35	-	-	-	-	-	-
**65**	spathulenol	1577	1570	85	-	-	1.1	-	-	0.20	0.33	0.25
**66**	caryophyllene oxide	1582	1575	88	13.42	3.88	2.76	4.48	15.28	10.50	3.23	15.96
**67**	humulene epoxide II	1608	1600	88	1.11	1.05	-	0.48	1.76	0.38	0.42	1.74
**68**	1.10-di-*epi*-cubenol	1618	1604	86	-	1.04	-	-	0.15	-	0.18	0.46
**69**	*allo*-epoxide aromadendrene	1639	1630	80	-	-	0.05	-	-	-	-	0.18
**70**	khusilal	1647	1639	80	0.41	0.54	-	-	0.11	-	-	0.38
**71**	pogostol	1651	1643	80	-	0.54	-	-	-	-	-	-
**72**	(*E*)-14-hydroxy-9-*epi*-caryophyllene	1668	1660	80	-	-	-	0.16	0.50	-	-	-
**73**	*cis*-thujopsenal	1708	1698	*	-	0.15	-	-	-	-	-	-
Monoterpene Hydrocarbons					29.98	52.86	34.66	1.53	25.39	59.79	24.37	16.21
Oxygenated Monoterpenes					44.63	9.99	49.94	86.44	45.93	25.08	40.02	34.24
Sesquiterpene Hydrocarbons					6.31	26.29	8.89	3.89	5.10	3.07	30.49	22.52
Oxygenated Sesquiterpenes					15.49	9.05	3.96	5.26	17.95	11.14	4.23	19.49
Total Identified					96.41	98.19	97.45	97.12	94.37	99.08	99.11	92.46

^a^ Compounds identified comparing the substance mass spectra with NIST 14 data base, literature [26] and filtered by the retention index (LTPRI); ^b^ LTPRI: Linear temperature programmed retention indices; ^c^ Exp.: LTPRI experimental obtained by the injection of a homologous series of C8–C20 *n*-alkanes using the Van den Dool and Kratz equation [27]; ^d^ Lit.: LTPRI obtained from literature [26]; ^e^ Similarity of compounds based on NIST 14 database (National Institute of Standards—Gaithersburg, MD, USA). * Compounds identified based on literature [26]; ^f^ Concentration of the metabolites were obtained by area normalization.

## Data Availability

Plants of *Lippia alba* were used in this study. All collected material belongs to the Germplasm Bank of the Agronomic Institute, Campinas, SP, Brazil.

## Sample Availability

Samples of the compounds are not available from the authors.

## References

[1] Do Nascimento Soares C.H., de Mello Campos I., dos Santos Amorim G.T., do Carmo M.G.F., de Almeida Chaves D.S., de Souza M.A.A. (2019). Selection of genotypes (citral chemotype) of *Lippia alba* (Mill.) N. E. Brown regarding seasonal stability of the essential oils chemical profile. Ind. Crops Prod..

[2] Rufino E.R., Siqueira W.J., Marques M.O.M., Colombo C.A., Azevedo Filho J.A., Martins A.L.M. (2012). Selection of new clones of linalool chemotype from genetic recombination in *Lippia alba*. Bragantia.

[3] Vale T.G., Matos F.J.A., De Lima T.C.M., Viana G.S.B. (1999). Behavioral effects of essential oils from *Lippia alba* (Mill.) N.E. Brown chemotypes. J. Ethnopharmacol..

[4] Herbia Cosmético Orgânico *Lippia alba*, Verbena Brasileira. http://herbia.com.br/produtos/lippia-alba/.

[5] Yamamoto P.Y., Colombo C.A., Azevedo Filho J.A., Lourenção A.L., Marques M.O.M., Morais G.D.D.S., Chiorato A.F., Martins A.L.M., Siqueira W.J. (2008). Performance of ginger grass (*Lippia alba*) for traits related to the production of essential oil. Sci. Agric..

[6] Montanari R.M., Barbosa L.C.A., Demuner A.J., Silva C.J., Carvalho L.S., Andrade N.J. (2011). Chemical composition and antibacterial activity of essential oils from Verbenaceae species: Alternative sources of (*E*)-Caryophyllene and Germacrene D. Quim. Nova.

[7] Rukhsar A.D., Mushtaq A., Sanjay K., Monica R. (2015). Agriculture germplasm resources: A tool of conserving diversity. Sci. Res. Essays.

[8] Machado L.C., Oliveira V.C., Paraventi M.D., Cardoso R.N.R., Martins D.S., Ambrósio C.E. (2016). Maintenance of Brazilian Biodiversity by germplasm bank. Pesqui. Vet. Bras..

[9] Nass L.L., Sigrist M.S., Ribeiro C.S.d.C., Reifschneider F.J.B. (2012). Genetic resources: The basis for sustainable and competitive plant breeding. Crop Breed. Appl. Biotechnol..

[10] Datta A. (2013). Genetic engineering for improving quality and productivity of crops. Agric. Food Secur..

[11] Ming L.C., Erhlet P., Siqueira W.J., Marques M.O.M. (2016). Produtos Naturais Bioativos, Chapter 2: Botânica, Fitoquímica, Cultivo e Melhoramento Genético de Lippia alba (Mill.) N.E.Br. (Verbenaceae).

[12] Schocken N.R.L. (2007). Obtenção de quimiotipos híbridos de *Lippia alba* (Mill.) N. E. Brown. Master Diss. (Master Trop. Subtrop. Agric. Conc. Area Plant Breeding).

[13] Ortega-Cuadros M., de Guevara E.E.A., Castillo A.D.M., Castañeda C.G., Amarís G.C., Tofiño-Rivera A.P. (2020). Essential oils biological activity of the shrub *Lippia alba* (Verbenaceae). Rev. Biol. Trop..

[14] Lorenzo D., Paz D., Davies P., Vila R., Caigueral S., Dellacassa E. (2001). Composition of a new essential oil type of *Lippia alba* (Mill.) N.E. Brown from Uruguay. Flavour Fragr. J..

[15] Welke J.E., Zini C.A. (2011). Comprehensive two-dimensional gas chromatography for analysis of volatile compounds in foods and beverages. J. Braz. Chem. Soc..

[16] Baschieri A., Ajvazi M.D., Tonfack J.L.F., Valgimigli L., Amorati R. (2017). Explaining the antioxidant activity of some common non-phenolic components of essential oils. Food Chem..

[17] Facanali R., Marques M.O.M., Hantao L.W. (2020). Metabolic profiling of *Varronia curassavica* Jacq. Terpenoids by flow modulated two-dimensional gas chromatography coupled to mass spectrometry. Separations.

[18] Paiva A.C., Oliveira D.S., Hantao L.W. (2019). A bottom-up approach for data mining in bioaromatization of beers using flow-modulated comprehensive two-dimensional gas chromatography/mass spectrometry. Separations.

[19] Paiva A.C., Hantao L.W. (2020). Exploring a public database to evaluate consumer preference and aroma profile of lager beers by comprehensive two-dimensional gas chromatography and partial least squares regression discriminant analysis. J. Chromatogr. A.

[20] Hantao L.W., Aleme H.G., Passador M.M., Furtado E.L., de Ribeiro F.A.L., Poppi R.J., Augusto F. (2013). Determination of disease biomarkers in *Eucalyptus* by comprehensive two-dimensional gas chromatography and multivariate data analysis. J. Chromatogr. A.

[21] Hantao L.W., Toledo B.R., De Lima Ribeiro F.A., Pizetta M., Pierozzi C.G., Furtado E.L., Augusto F. (2013). Comprehensive two-dimensional gas chromatography combined to multivariate data analysis for detection of disease-resistant clones of *Eucalyptus*. Talanta.

[22] Guo J., Li J., Yang X., Wang H., He J., Liu E., Gao X., Chang Y.X. (2020). A Metabolomics Coupled With Chemometrics Strategy to Filter Combinatorial Discriminatory Quality Markers of Crude and Salt-Fired *Eucommiae Cortex*. Front. Pharmacol..

[23] Boccard J., Rudaz S. (2020). Analysis of Metabolomics Data—A Chemometrics Perspective.

[24] Lim Ah Tock M.J., Kamatou G.P.P., Combrinck S., Sandasi M., Viljoen A.M. (2020). A chemometric assessment of essential oil variation of three *Salvia* species indigenous to South Africa. Phytochemistry.

[25] Allenspach M., Valder C., Flamm D., Grisoni F., Steuer C. (2020). Verification of chromatographic profile of primary essential oil of *Pinus sylvestris* L. Combined with chemometric analysis. Molecules.

[26] Adams R.P. (2017). Identification of Essential Oil Components by Gas Chromatography/Mass Spectroscopy.

[27] Van den Dool H., Kratz P.D. (1963). A generalization of the retention index system including liner temperature programmed gas-liquid partition chromatography. J. Chromatogr. A.

[28] Blank A.F., Camêlo L.C.A., Arrigoni-Blank M.F., Pinheiro J.B., Andrade T.M., Niculau E.S., Alves P.B. (2015). Chemical diversity in *Lippia alba* (Mill.) N. E. Brown Germplasm. Sci. World J..

[29] Da Silva A.C.C., Barbosa F.G., Mafezoli J., Oliveira M.C.F., Oliveira T.F. (2017). HS-SPME as an efficient tool for discriminating chemotypes of *Lippia alba* (Mill.) N. E. Brown. Quim. Nov..

[30] Hennebelle T., Sahpaz S., Dermont C., Joseph H., Bailleul F. (2006). The essential oil of *Lippia alba*: Analysis of samples from french overseas departments and review of preview works. Chem. Biodivers..

[31] Ricciardi G., Ciccio J.F., Ocampo R., Lorenzo D., Ricciardi A., Bandoni A., Dellacassa E. (2009). Chemical Variability of Essential Oils of *Lippia alba* (Miller) N. E. Brown growing in Costa Rica and Argentina. Nat. Prod. Commun..

[32] Dos Marques C.T.S., Gama E.V.S., da Silva F., Teles S., Caiafa A.N., Lucchese A.M. (2018). Improvement of biomass and essential oil production of *Lippia alba* (Mill) N.E. Brown with green manures in succession. Ind. Crops Prod..

[33] Oliveira D.R., Leitão G.G., Santos S.S., Bizzo H.R., Lopes D., Alviano C.S., Alviano D.S., Leitão S.G. (2006). Ethnopharmacological study of two *Lippia* species from Oriximiná, Brazil. J. Ethnopharmacol..

[34] Jannuzzi H., Mattos J.K.A., Vieira R.F., Silva D.B., Bizzo H.R., Gracindo L.A.M. (2010). Avaliação agronômica e identificação de quimiotipos de erva cidreira no Distrito Federal. Hortic. Bras..

[35] Pistelli L., Ferri B., Cioni P.L., Koziara M., Agacka M., Skomra U. (2018). Aroma profile and bitter acid characterization of hop cones (*Humulus lupulus* L.) of five healthy and infected Polish cultivars. Ind. Crops Prod..

[36] Behr A., Johnen L. (2009). Myrcene as a natural base chemical in sustainable chemistry: A critical review. ChemSusChem.

[37] Evergetis E., Koulocheri S.D., Haroutounian S.A. (2015). Exploitation of Apiaceae Family plants as valuable renewable source of essential oils containing crops for the production of fine chemicals: Part II. Ind. Crops Prod..

[38] Salanţă L., Tofană M., Socaci S., Mudura E., Pop C., Pop A., Fărcaş A. (2015). Evaluation and comparison of aroma volatile compounds in hop varieties grown in Romania. Rom. Biotechnol. Lett..

[39] Yang X., Li S., Xia J., Song J., Huang K., Li M. (2015). Novel renewable resource-based UV-curable copolymers derived from myrcene and tung oil: Preparation, characterization and properties. Ind. Crops Prod..

[40] Rufino A.T., Ribeiro M., Sousa C., Judas F., Salgueiro L., Cavaleiro C., Mendes A.F. (2015). Evaluation of the anti-inflammatory, anti-catabolic and pro-anabolic effects of E-caryophyllene, myrcene and limonene in a cell model of osteoarthritis. Eur. J. Pharmacol..

[41] Jansen C., Shimoda L.M.N., Kawakami J.K., Ang L., Bacani A.J., Baker J.D., Badowski C., Speck M., Stokes A.J., Small-Howard A.L. (2019). Myrcene and terpene regulation of TRPV1. Channels.

[42] Tavares E.S., Julião L.S., Lopes D., Bizzo H.R., Lage C.L.S., Leitão S.G. (2005). Análise do óleo essencial de folhas de três quimiotipos de *Lippia alba* (Mill.) N. E. Br. (Verbenaceae) cultivados em condições semelhantes. Braz. J. Pharmacogn..

[43] Salehi B., Upadhyay S., Orhan I.E., Jugran A.K., Jayaweera S.L.D., Dias D.A., Sharopov F., Taheri Y., Martins N., Baghalpour N. (2019). Therapeutic potential of α-and β-pinene: A miracle gift of nature. Biomolecules.

[44] Ueno H., Shimada A., Suemitsu S., Murakami S., Kitamura N., Wani K., Matsumoto Y., Okamoto M., Ishihara T. (2019). Attenuation Effects of Alpha-Pinene Inhalation on Mice with Dizocilpine-Induced Psychiatric-Like Behaviour. Evid. Based Complement. Altern. Med..

[45] Da Silva A.C.R., Lopes P.M., de Azevedo M.M.B., Costa D.C.M., Alviano C.S., Alviano D.S. (2012). Biological Activities of α-Pinene and β-Pinene Enantiomers. Molecules.

[46] Matsuo A.L., Figueiredo C.R., Arruda D.C., Pereira F.V., Scutti J.A.B., Massaoka M.H., Travassos L.R., Sartorelli P., Lago J.H.G. (2011). α-pinene isolated from *Schinus terebinthifolius* Raddi (Anacardiaceae) induces apoptosis and confers antimestastatic protection in a melanoma model. Biochem. Biophys. Res. Commun..

[47] Rufino A.T., Ribeiro M., Judas F., Salgueiro L., Lopes M.C., Cavaleiro C., Mendes A.F. (2014). Anti-inflammatory and chondroprotective activity of (+)-α-pinene: Structural and enantiomeric selectivity. J. Nat. Prod..

[48] Pinheiro M.D.A., Magalhães R.M., Torres D.M., Cavalcante R.C., Mota F.S.X., Coelho E.M.A.O., Moreira H.P., Lima G.C., Araújo P.C.D.C., Cardoso J.H.L. (2015). Gastroprotective effect of alpha-pinene and its correlation with antiulcerogenic activity of essential oils obtained from *Hyptis* species. Pharmacogn. Mag..

[49] Peixoto M.G., Bacci L., Fitzgerald Blank A., Araújo A.P.A., Alves P.B., Silva J.H.S., Santos A.A., Oliveira A.P., da Costa A.S., Arrigoni-Blank M.d.F. (2015). Toxicity and repellency of essential oils of *Lippia alba* chemotypes and their major monoterpenes against stored grain insects. Ind. Crops Prod..

[50] Bouzenna H., Hfaiedh N., Giroux-Metges M.A., Elfeki A., Talarmin H. (2017). Biological properties of citral and its potential protective effects against cytotoxicity caused by aspirin in the IEC-6 cells. Biomed. Pharmacother..

[51] Shi C., Song K., Zhang X., Sun Y., Sui Y., Chen Y., Jia Z., Sun H., Sun Z., Xia X.X. (2016). Antimicrobial activity and possible mechanism of action of citral against *Cronobacter sakazakii*. PLoS ONE.

[52] Cai R., Hu M., Zhang Y., Niu C., Yue T., Yuan Y., Wang Z. (2019). Antifungal activity and mechanism of citral, limonene and eugenol against *Zygosaccharomyces rouxii*. LWT.

[53] De Oliveira E.R., Alves D.S., Carvalho G.A., de Oliveira B.M.R.G., Aazza S., Bertolucci S.K.V. (2018). Toxicity of *Cymbopogon flexuosus* essential oil and citral for *Spodoptera frugiperda*. Cienc. Agrotecnol..

[54] Chaouki W., Leger D.Y., Liagre B., Beneytout J.L., Hmamouchi M. (2009). Citral inhibits cell proliferation and induces apoptosis and cell cycle arrest in MCF-7 cells. Fundam. Clin. Pharmacol..

[55] Shen Y., Sun Z., Guo X. (2015). Citral inhibits lipopolysaccharide-induced acute lung injury by activating PPAR-γ. Eur. J. Pharmacol..

[56] Nishijima C.M., Ganev E.G., Mazzardo-Martins L., Martins D.F., Rocha L.R.M., Santos A.R.S., Hiruma-Lima C.A. (2014). Citral: A monoterpene with prophylactic and therapeutic anti-nociceptive effects in experimental models of acute and chronic pain. Eur. J. Pharmacol..

[57] De Barros F.M.C., De Zambarda E.O., Heinzmann B.M., Mailmann C.A. (2009). Seasonal variability and terpenoid biosynthesis of the essential oil of *Lippia alba* (Mill.) N. E. Brown (Verbenaceae). Quim. Nova.

[58] Batista P.A., De Paula Werner M.F., Oliveira E.C., Burgos L., Pereira P., Da Silva Brum L.F., Story G.M., Santos A.R.S. (2010). The antinociceptive effect of (-)-linalool in models of chronic inflammatory and neuropathic hypersensitivity in mice. J. Pain.

[59] Hu J., Liu S., Deng W. (2020). Dual responsive linalool capsules with high loading ratio for excellent antioxidant and antibacterial efficiency. Colloids Surfaces B Biointerfaces.

[60] Ma J., Xu H., Wu J., Qu C., Sun F., Xu S. (2015). Linalool inhibits cigarette smoke-induced lung inflammation by inhibiting NF-κB activation. Int. Immunopharmacol..

[61] Peana A.T., D’Aquila P.S., Panin F., Serra G., Pippia P., Moretti M.D.L. (2002). Anti-inflammatory activity of linalool and linalyl acetate constituents of essential oils. Phytomedicine.

[62] Rodenak-Kladniew B., Castro A., Stärkel P., De Saeger C., García de Bravo M., Crespo R. (2018). Linalool induces cell cycle arrest and apoptosis in HepG2 cells through oxidative stress generation and modulation of Ras/MAPK and Akt/mTOR pathways. Life Sci..

[63] Pereira I., Severino P., Santos A.C., Silva A.M., Souto E.B. (2018). Linalool bioactive properties and potential applicability in drug delivery systems. Colloids Surfaces B Biointerfaces.

[64] Juergens U.R. (2014). Anti-inflammatory Properties of the Monoterpene 1.8-cineole: Current Evidence for Co-medication in Inflammatory Airway Diseases. Drug Res..

[65] Amorati R., Foti M.C., Valgimigli L. (2013). Antioxidant activity of essential oils. J. Agric. Food Chem..

